# Tuning the Mechanical Properties of Polymer Nanocomposites Filled with Grafted Nanoparticles by Varying the Grafted Chain Length and Flexibility

**DOI:** 10.3390/polym8090270

**Published:** 2016-08-25

**Authors:** Zixuan Wang, Zijian Zheng, Jun Liu, Youping Wu, Liqun Zhang

**Affiliations:** 1 Key Laboratory of Beijing City on Preparation and Processing of Novel Polymer Materials, Beijing University of Chemical Technology, Beijing 100029, China; wangzixuan@vip.163.com (Z.W.); zhengzijian2008@126.com (Z.Z.); wuyp@mail.buct.edu.cn (Y.W.); 2 Beijing Engineering Research Center of Advanced Elastomers, Beijing 100029, China; 3 Engineering Research Center of Elastomer Materials on Energy Conservation and Resources, Ministry of Education, Beijing 100029, China; 4 State Key Laboratory of Organic-Inorganic Composites, Beijing University of Chemical Technology, Beijing 100029, China

**Keywords:** self-assembly, polymer-grafted NPs, polymer nanocomposites

## Abstract

By employing coarse-grained molecular dynamics simulation, we simulate the spatial organization of the polymer-grafted nanoparticles (NPs) in homopolymer matrix and the resulting mechanical performance, by particularly regulating the grafted chain length and flexibility. The morphologies ranging from the agglomerate, cylinder, sheet, and string to full dispersion are observed, by gradually increasing the grafted chain length. The radial distribution function and the total interaction energy between NPs are calculated. Meanwhile, the stress–strain behavior of each morphology and the morphological evolution during the uniaxial tension are simulated. In particular, the sheet structure exhibits the best mechanical reinforcement compared to other morphologies. In addition, the change of the grafted chain flexibility to semi-flexibility leads to the variation of the morphology. We also find that at long grafted chain length, the stress–strain behavior of the system with the semi-flexible grafted chain begins to exceed that of the system with the flexible grafted chain, attributed to the physical inter-locking interaction between the matrix and grafted polymer chains. A similar transition trend is as well found in the presence of the interfacial chemical couplings between grafted and matrix polymer chains. In general, this work is expected to help to design and fabricate high performance polymer nanocomposites filled with grafted NPs with excellent and controllable mechanical properties.

## 1. Introduction

Polymer nanocomposites (PNCs) filled with various kinds of nanoparticles (NPs) have been a hot topic in academia and industry because of the promising mechanical, electrical, and thermal properties;gas permeability; and so on [1,2,3]. This is realized by combining the functionality of NPs with the good processability of polymers. In most cases, it is commonly accepted that a homogeneous dispersion of NPs is needed to achieve improved physical properties such as reduced permeability and enhanced mechanical strength. Conversely, to obtain excellent thermal and electrical conductivity, precise assembly and ordering of NPs to form a continuous network should be developed. Therefore, to effectively tailor the properties of PNCs inspires us to establish some strategies to finely manipulate the spatial arrangement and localization of NPs in polymer matrices [4,5,6].

One effective approach is to uniformly graft the NPs with short polymer chains [3,7,8]. For instance, Akcora et al. [9] have observed that the grafted spherical NPs can robustly self-assemble into a variety of anisotropic superstructures in the corresponding homopolymer matrix, attributed to the competition between the energy gain when the NPs approach each other and the entropy of distorting the grafted polymers. Kumar et al. [8] also pointed out that using the polymer grafted NPs is effective to control the spatial dispersion of the NPs in the polymer matrices, which is ignited by the development of the synthesis techniques to controllably functionalize NPs with polymer chains. For example, Chen-Yang et al. [10] have successfully prepared precisely tailored polystyrene (PS)-grafted NPs, including those with varied shapes and sizes of cores but identical PS shells, and NPs with fixed shapes but varied PS shells, which are considered ideal model NPs to probe the dynamics and reinforced mechanism of PNCs. It as well commonly accepted that the morphological behavior of grafted NPs in polymer matrices is similar to the block copolymers or amphiphiles in selective solvents [11]. Meanwhile, Green PF [12] reviewed the morphological phase diagrams defining the miscible, incompatible or partially miscible regions, by systematically considering the effects of the nanoparticle curvature, the chain grafting density and length, the length of the polymer matrix and the thermodynamic (Flory–Huggins) interaction parameter between the host and grafted chains. The effects of the grafted chain flexibility [13], the polymer matrix bidispersity with the equal number of short and long chains [14] on the morphology of the grafted NPs filled PNCs are also studied. Meanwhile, through the microscopic polymer reference interaction site model integral equation theory, Jayaraman et al. [15] have calculated the potential of mean force (PMF) between grafted NPs in the homopolymer matrix. Pryamtisyn et al. [16] have predicted that the polymer grafted NPs can readily form the self-assembled structures when the nanoparticle size is comparable to the radius of the gyration of the polymer brushes. By extending to much higher loading of NPs, Ginzburg V.V. [17] adopted the combined self-consistent field theory (SCFT)-density functional theory(DFT) approach to study the dependence of the morphology on the length and density of grafted chains, finding a new morphology of bundles of wires compared to the lower loading, and this method can be also applicable for some other cases that the matrix or the ligand is a kind of polymer blend or block copolymer.

However, althoughplenty of previous studies have been carried out to study the effects of the structural parameters on the spatial distribution and organization of the end-grafted NPs in the polymer matrices, little efforts have been devoted to establish the relation between the morphology and the resulting mechanical properties. Through the rheology analysis of polymer-grafted NPs filled polymer nanocomposites, Moll et al. [3] found that the formation of a transient, long-lived, percolating polymer–NPs network with the NPs serving as the network junctions contributes to the maximum mechanical reinforcement. Experimentally, they also studied the effect of the shear flow on the morphological evolution of the assembles of grafted spherical NPs [18]. In particular, Riggleman et al. [19] have simulated the effect of the curvature of the NPs and the grafting density on the mechanical properties of PNCs, finding that the elastic constants and yield properties are enhanced nearly uniformly, while the strain hardening modulus is weakly affected.

In this work, we fix the loading of the NPs and the number of the grafted polymer chains, and we aim to simulate all the possible morphologies formed by the grafted NPs in the same homopolymer matrix and the resulting mechanical properties by particularly varying the grafted chain length and flexibility, in hopes of providing scientific guidance for the fabrication of PNCs with excellent and tunable mechanical properties with significant practical applications.

## 2. Model and Simulation Techniques

In this work, we adopt a coarse-grained model to model the polymer chains such as the bead-spring model, in which each bead corresponds to 3–6 bonds of a realistic polymer chain. The surface of the modeled NPs is grafted evenly with six short polymer oligomers, which are chemically identical with the polymer matrix. Each matrix polymer chain contains 100 beads, and the number of matrix polymer chains is 300. It is noted that the grafting density is fixed, while we vary the grafted chain length *L*_g_ from 2 to 36 beads.

**Figure 1:**
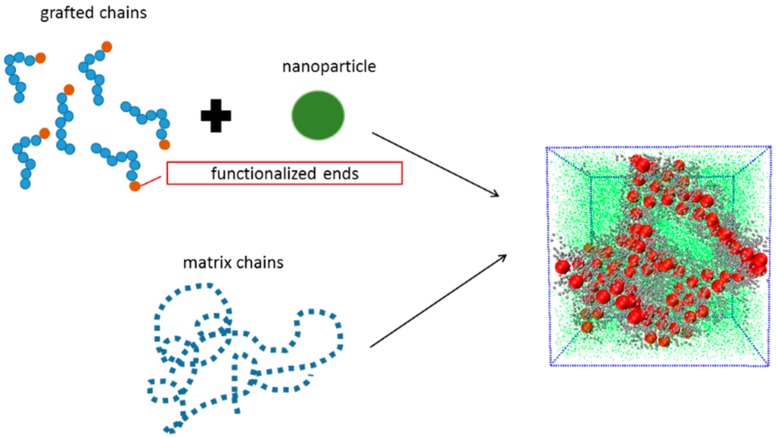
The module of the construction of the nanocomposites. Note that on the right side in the simulation box, the NPs are denoted by the red spheres, the grafted chain beads are denoted by the silver spheres, and matrix chain beads are denoted by the green dots.

To graft the short polymer oligomers onto the surface of each NP, we follow our previous approach [20] as follows: we introduce the virtual points on the surface of the NP, leading to the NPs composed of a hard sphere with a diameter of σ,where σ is the distance unit, and 96 virtual points evenly distributed on the surface of each hard sphere , and the type of beads is shown in Table 1. The reason to introduce these virtual points on the surface of the NPs is to fix the grafted points, so that these chains can be evenly grafted. These virtual points are similar to the chemical functionalization groups. Every virtual point is bonded with every other point and with the central core, which makes them move in the simulation box as a whole body. Our system contains 100 NPs, with its volume fraction approximately equal to 26.8%. To better illustrate our nanocomposite model, we hereby utilize schematics to explain the setup process, with *L*_g_ = 8, as is seen in Figure 1.

The non-bonded interactions are modeled by the expanded truncated and shifted Lennard–Jones (LJ) interaction, which include the polymer–polymer, polymer–nanoparticle, and nanoparticle–nanoparticle interactions, given by

(1) $$U=\{\begin{array}{ll} 4\varepsilon \left[{\left(\frac{\sigma }{r-\Delta }\right)}^{12}-{\left(\frac{\sigma }{r-\Delta }\right)}^{6}\right]+C & \;r<r_{cutoff}+\Delta \\ 0 & r\geq r_{cutoff}+\Delta \end{array}$$

where ε is the pair interaction energy parameter, *r* is the distance between two interaction sites, while σ is the distance unit, and Δ is the effect of excluded volume of different interaction sites in the LJ function. Hence, the actual cutoff distance is the sum of *r*_cutoff_ and Δ. For polymer-nanoparticle interactions *U*_np_ and nanoparticle-nanoparticle interactions *U*_nn_, Δ is $R_{NP}-\sigma /2$ and $2R_{NP}-\sigma$, respectively. Here, *C* is a constant that guarantees the condition that when $r=r_{\text{cutoff}}+\Delta$, the value of U equals to zero. In our simulation, the interaction energy parameter of the nanoparticle–nanoparticle, i.e., $\varepsilon_{nn}$ is set to be 10.0, whereas the parameter of the nanoparticle–polymer, $\varepsilon_{np}$ and polymer–polymer, $\varepsilon_{pp}$, are both set to be 1.0. Since we are not studying a specific polymer chain, we hereby simplify our parameters by setting both σ and ε equal to unit. Therefore, all the simulated parameters are dimensionless. The LJ interaction energy is cut off at different distances to model the attractive or repulsive interactions. The $r_{\text{cutoff}}$ stands for the distance at which the interaction is truncated and shifted so that the energy is equal to zero at this distance. The polymer–polymer interaction and polymer–nanoparticle interaction parameters are both set to be $r_{\text{cutoff}}=2\times 2^{1/6}\sigma$ to represent a short-ranged attraction, whereas the nanoparticle–nanoparticle parameter is set to be $r_{\text{cutoff}}=2.5\sigma$ to model a long-ranged attraction.

The bonded interaction between the neighbor beads along a polymer chain is modeled as the harmonic potential:

(2) $$E_{\text{bond}}=k{\left(r-r_{0}\right)}^{2}$$

where k is a constant demonstrating the strength of a bond and the value is k=1000.0, and $r_{0}$ is the equilibrium bond distance which equals to $r_{0}=1.0$, ensuring a certain stiffness while preventing the polymer beads becoming overlapped with each other.

T=5.0 $\rho^{*}=0.85$ Then, the NPT ensemble is adopted, where the temperature isfixed at T=5.0 and the pressure is fixed at 1 atm for all situations by using the Nose–Hoover temperature thermostat and pressure barostat, which means the NPT ensemble is to compress the system volume and to reduce the number density of polymer matrix, keeping it around $\rho^{*}=0.85$, corresponding to the density of the polymer melt. The periodic boundary condition in the three directions is applied, and a velocity-Verlet algorithm is applied with a timestep δt=0.001τ, in which τ stands for time unit in coarse-grained model. After this, we graft all end functionalized short polymer chains onto virtual points, i.e., the surface of the NPs. Once a grafted chain approaches any random virtual point, a chemical bond is formed between these two beads and therefore the grafted chain is fixed onto the surface. It is noted that this new chemical bonding is as well modeled by the same harmonic potential as other bonded potentials. One grafting site can only be bonded by one grafting chain. After a rather amount of the simulation time, each NP is evenly grafted with six chains. Finally, the NVT ensemble is adopted, where the temperature is kept at T=5.0, and the volume of the simulation box is fixed throughout the whole simulation process. The aim of the NVT ensemble is to further equilibrate the systems so that each chain has moved at least $2R_{g}$.

To study the tensile deformation, we increase the length of the simulation box in the y direction at a fixed engineering strain, while the box lengths in the x and z directions are reduced simultaneously to maintain the volume of the simulation box during the deformation process. The tensile strain rate is defined as follows: $l=\left(L_{y}(t)-L_{y}(0)\right)/L_{y}(0)=0.015/\tau$. In this case, $L_{y}$ stands for the length of simulation box in the y direction. The average tensile stress in the y direction is calculated from each dimension of the stress variation: $\sigma_{t}=(1+\mu )(-P_{yy}+P)\approx 3(-P_{yy}+P)/2$, in which case $P=\Sigma_{i}(P_{ii}/3)$ is the composed pressure. The parameter μ is Poisson’s ratio, which is equal to0.5 in our simulation. For our tensile tests, unless otherwise stated, the maximum strain is 300% larger than the initial length, and thus we run 2 × 10^6^ runs for each tensile test.

All MD runs are carried out through the large scale atomic/molecular massively parallel simulator (LAMMPS), which is developed by Sandia National Laboratories. For all cases, we make sure that each chain has transported at least $2R_{g}$, where $R_{g}$ is the root mean square radius of gyration of polymer chains, to obtain well-equilibrated systems. More detailed simulation techniques can be found in our previously published work [20,21,22]. To make our results more reliable and accurate, we repeat each trial for fivetimes, and then acquire an average value for each variable such as non-bonded interaction energies $U_{nn}$ and radial distribution function g(r). For stress–strain curves, we also adopted fivetrials, and then the stress is averaged for fivetrials in order to obtain final plots.

## 3. Results and Discussion

### 3.1. Effect of the Grafted Polymer Chain Length

**Figure 2:**
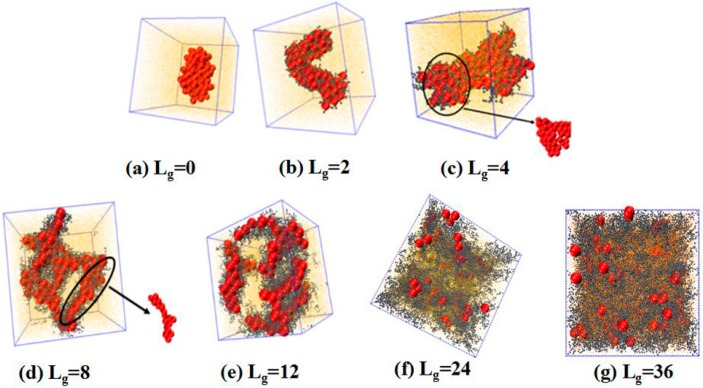
The formed structures via the self-assembly of the spherical nanoparticles (NPs) grafted with various lengths of polymer chains in the polymer matrix: the lengths of the grafted short polymer chains are as follows: (**a**) $L_{g}=0$; (**b**) $L_{g}=2$; (**c**) $L_{g}=4$; (**d**) $L_{g}=8$; (**e**) $L_{g}=12$; (**f**) $L_{g}=24$; and (**g**) $L_{g}=36$. Note that the number of the grafted polymer chains on one NP is fixed to be six, represented by the purple beads. The length of the polymer matrix is set to 100, represented by the yellow points. The NPs are denoted by the red spheres.

We firstly examined the self-assembly structures, potentially formed by NPs uniformly grafted with the same chains as the matrix polymer chains. Note that we fix the grafting density equal to 0.18, given by:

(3) $$\sigma_{grafting}=\frac{N}{S_{NP}}$$

where N denotes the number of grafting chains grafted on each nanoparticle, and $S_{NP}$ denotes the surface area of each nanoparticle, while the grafted chain length is varied as follows: $L_{g}=0$ (bare particles), $L_{g}=2$, $L_{g}=4$, $L_{g}=8$, $L_{g}=12$, $L_{g}=24$ and $L_{g}=36$. We place 100 NPs in the simulation box to observe the self-assembled structures. The simulated results are shown in Figure 2. Obviously, without any grafting, the bare NPs tend to aggregate into an isotropic cluster, because of the incompatibility between the NPs and the matrix polymer chains. In other cases, by gradually increasing the grafted chain length, a variety of structures are formed. For instance, when the grafted chains contain two beads ($L_{g}=2$), the NPs self-assemble to form a cylinder structure, which is changed to sheets with the length of the grafted chains $L_{g}=4$. Furthermore, a connected string structure and a bunch of short strings occur in the case of $L_{g}=8$ and $L_{g}=12$, respectively. These observations are in qualitative agreement with the work carried out by Akcora et al. [9]. When the grafted chain length increases to $L_{g}=24$, most NPs become separated with each other and dispersed, and when the grafted chain length is increased to $L_{g}=36$, all NPs become well dispersed in the polymer matrix. This observation is consistent with our previous work that NPs tend to become dispersed in the polymer matrix when they are densely grafted or grafted with long polymer chains [20]. The underlying mechanism results from the competition between the entropy contributed by the conformation of the grafted polymer chains, and enthalpy between the NPs. When the grafted chain length becomes long enough, the long grafted polymer chains repel the NPs apart, and meanwhile a strong interlocking occurs between the long grafted polymer chains and the matrix polymer chains. The formed structure represents the maximum contact between NPs via the un-shielded surfaces. Mahynski et al. [15] pointed out that different chain lengths lead to different configurations around NPs, that is, longer grafted chains lead to a larger area of coverage onto the surface of NPs, in analogy to the “patchy particle” offering the rest of the exposed surfaces to contact with each other. Thus, it is our speculation that when the NPs are grafted by relatively short chains such as $L_{g}=2$, the grafted chains can provide little coverage for the surface of the NPs, since the surface area of the NPs is 16 times greater than that of each polymer bead, offering opportunities for the NPs to attract with each other. However, in the cases of $L_{g}=8$ and $L_{g}=12$, a string structure is formed, whereas, for $L_{g}=4$, the sheet structure is more likely to form when the interaction energy between NPs is strong enough to overcome the entropy penalty of the grafted chains.

**Figure 3:**
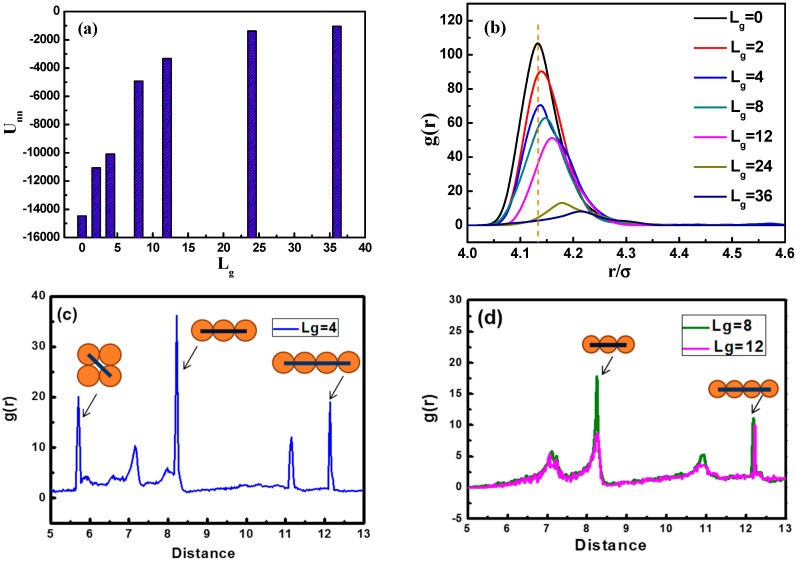
(**a**) The total interaction energy of NPs $U_{nn}$ as a function of the length of the grafted polymer chains $L_{g}$; (**b**) the radial distribution function of NPs g(r) for various lengths of the grafted polymer chains $L_{g}$; (**c**) the radial distribution function of NPs g(r) with grafted chain length $L_{g}=4$ (**blue**); and (**d**) the radial distribution function of NPs g(r) with grafting chain length $L_{g}=8$ (**green**) and $L_{g}=12$ (**pink**).

In Figure 3a, we calculate the total interaction energy between NPs as a function of the grafted chain length to reflect the aggregation state. It is clearly shown that the total interaction energy between NPs for $L_{g}=4$ is much larger than that of $L_{g}=8$ and $L_{g}=12$. This may serve as evidence that when $L_{g}$ is equal to fourpolymer beads, the NPs self-assemble into sheets instead of strings. It is evident that as the grafting chain length becomes longer, the total interaction energy between NPs becomes weaker. A weaker total interaction energy indicates a relatively good dispersion of NPs, as supported by the snapshots in Figure 2 As the grafted polymer chains become longer, the matrix polymer chains gradually become inter-locked with the grafted chains attributed to the attractive interaction between each other, leading to a better dispersion of NPs.

To better characterize the self-assembled structures, we use the radial distribution function (RDF), as shown in Figure 3b. It can be seen that the height of the first peak declines gradually with the increase of the grafted chain length. Note that the first peak appears at the distance of approximately 4.13σ, corresponding to the direct contact equilibrium distance between NPs. When the grafted chain length $L_{g}$ is equal to 36, the first peak almost disappears, implying that the NPs become well dispersed. From Figure 3c,d, we demonstrate the RDF plots of NPs with grafted chains of $L_{g}=4$, $L_{g}=8$, and $L_{g}=12$, at a distance from 5σ to 13σ. Figure 3c displays that three main peaks are located at a distance of 5.72σ, 8.09σ, and 12.13σ, separately. The diameter of each NP is 4σ, and if four NPs are arranged into a square-like structure, the length of the diagonal line is equal to $4\times \sqrt{2}\approx 5.6\sigma$, so the peak at the distance of around 5.72σ makes sense as NPs pack into a square-like form. This gives an evidence of the occurrence of the sheet network. Figure 3d displays situations concerning $L_{g}=8$ and $L_{g}=12$, where two main peaks located at a distance of 8σ and 12σ can be observed, and peaks at a distance of 4(n+1)σ imply the fact that two NPs are separated by the number of n NPs, forming a chain-like structure, exhibiting the string morphology.

**Figure 4:**
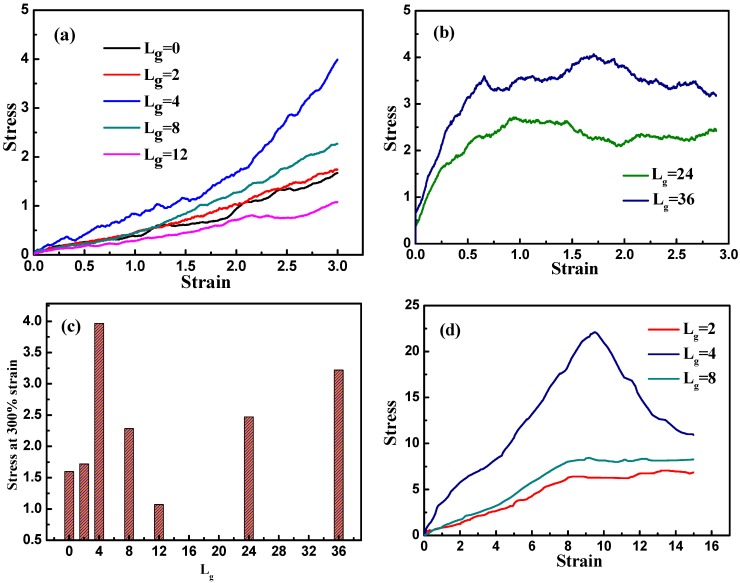
The stress–strain curves for systems filled with spherical NPs grafted with various chain lengths: (**a**) $L_{g}=0,2,4,8,12$; and (**b**) $L_{g}=24,36$; (**c**) The stress at 300% strain with respect to different grafted chain lengths $L_{g}$; (**d**) The stress–strain curves by performing a large tensile deformation for three systems with grafted chain lengths of $L_{g}=2$, $L_{g}=4$ and $L_{g}=8$.

We then investigate the tensile process of various self-assembled structures, expecting to find the most optimal structure that exhibits the best mechanical performance. We simulate all the systems by stretching the simulation box along the y direction while keeping the box volume constant. Figure 4a displays the stress–strain curves corresponding to the various grafted chain length. It is evident that as the self-assembly structure transforms from agglomeration and cylinder to sheet, the tensile stress is enhanced greatly, while the slope of the stress–strain curve declines gradually as the anisotropic structure turns into string. This clearly shows that among these self-assembly structures, the sheet structure displays the best mechanical property. When the length of the grafted chains is increased to $L_{g}=24$ and $L_{g}=36$, the stress–strain curve shows a stress plateau at a rather small strain (approximately 75%). This stress plateau is generated because of the blocking effect of well-dispersed nanoparticles, and thus instead of stretching the self-assembled structures formed by nanoparticles, the matrix undertakes most of the stress. As the interactions among matrix chains are relatively small, they are able to slide over and deform quite easily; consequently, the stress barely changes with the increase of strain. For better illustration, we examine the mechanical reinforcement by comparing the tensile stress at the strain equal to 300%, as is shown in Figure 4c. Again, it is further confirmed that the best tensile property occurs when the self-assembly structure of the grafted NPs form sheets, compared to other morphologies.

**Figure 5:**
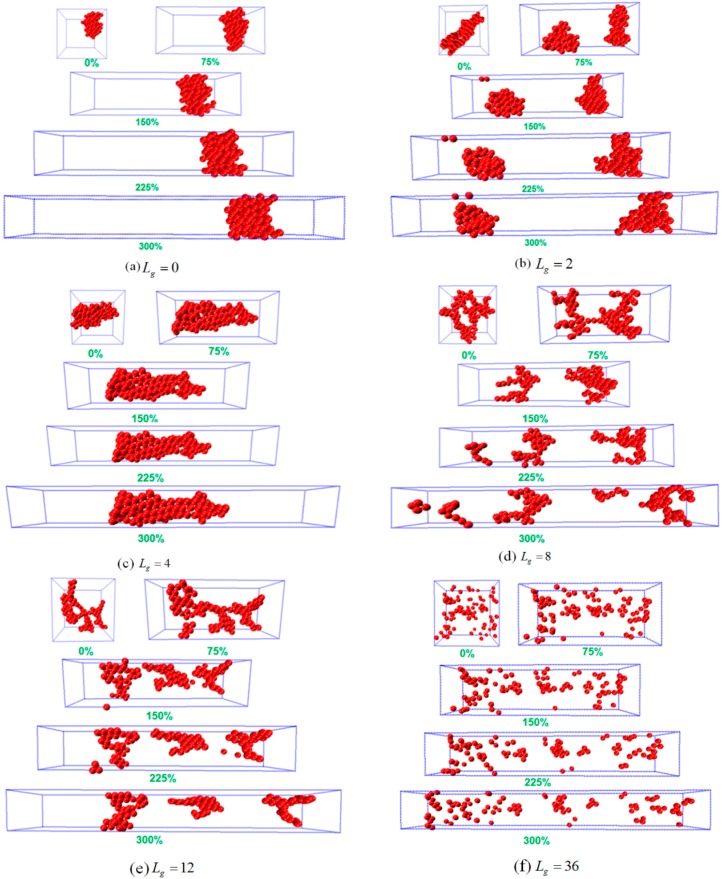
Snapshots of the uniaxial tension at five typical strains (0%, 75%, 150%, 225% and 300%) for various lengths of the grafted polymer chains: (**a**) $L_{g}=0$; (**b**) $L_{g}=2$; (**c**) $L_{g}=4$; (**d**) $L_{g}=8$; (**e**) $L_{g}=12$; and (**f**) $L_{g}=36$.

To analyze the possible reason of why the sheet structure endows the best mechanical performance, we probe the snapshots during the tensile process as shown in Figure 5. For $L_{g}=0$, the agglomerate formed by the bare NPs remains intact during the tensile deformation, indicating no breakage because of the strong attractive interaction between NPs. Similar phenomenon is observed for $L_{g}=2$, in which case the cylinder structure is initially broken into two small clusters, and then the clusters flow and align along the tensile direction. For $L_{g}=0$ and $L_{g}=2$, it can be seen that both systems exhibit an almost linear stress–strain curve, which explains the fact that the polymer matrix plays a dominant role during the tensile process. Furthermore, when the self-assembly structure transforms into sheets, and this structure gradually breaks and stretches into a rectangle with a longer side on the y direction. This observation implies that the percolating network itself undertakes a large part of the tensile force to enhance its mechanical property. In addition, when the grafted chain length is increased to $L_{g}=8$ and $L_{g}=12$, the string structure formed by the grafted NPs initially break, and then coarsen locally into small clusters, followed by the alignment and orientation along the tensile direction. For the sheet and string structures formed by the self-assembled grafted NPs, it is noted that even though the NPs percolating network plays a dominant role in these two cases, the string structure contributes less to the mechanical reinforcement compared to the sheet structure, attributed to much weaker interaction between the grafted NPs for the string structure. While for the dispersed states of $L_{g}=24$ and 36, during the tensile deformation the NPs do not show any phase transition, except for a slight agglomeration during the tensile process. However, a strong physical inter-locking occurs between the grafted and matrix polymer chains, in the case of long grafted polymer chains, which could lead to the increase of the mechanical reinforcement.

**Figure 6:**
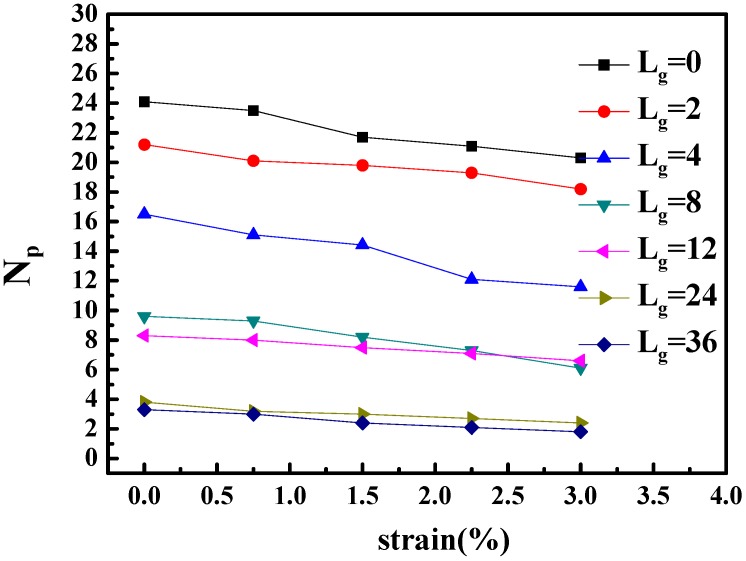
The number of neighbor particles $N_{P}$ with regard to strain when lengths of grafted chains are different.

To better identify that the microscopic origin of the stress is from the breakage of clusters, we calculated the shape deformation and cluster size of nanoparticles during the extension. The variation of the number of clusters and the number of nanoparticles per cluster are listed in Table 2. In addition, the change of the number of neighbor nanoparticles within the distance of 5.5σ (radius excluded) with regard of time evolution is illustrated in Figure 6. It can be seen that with the increase of strain, the cluster breaks into smaller pieces, and within the same cluster, the distance between particles become significantly smaller. This means that the clusters, i.e., the nanoparticle structures undertake the major part of the tensile stress.

**Figure 7:**
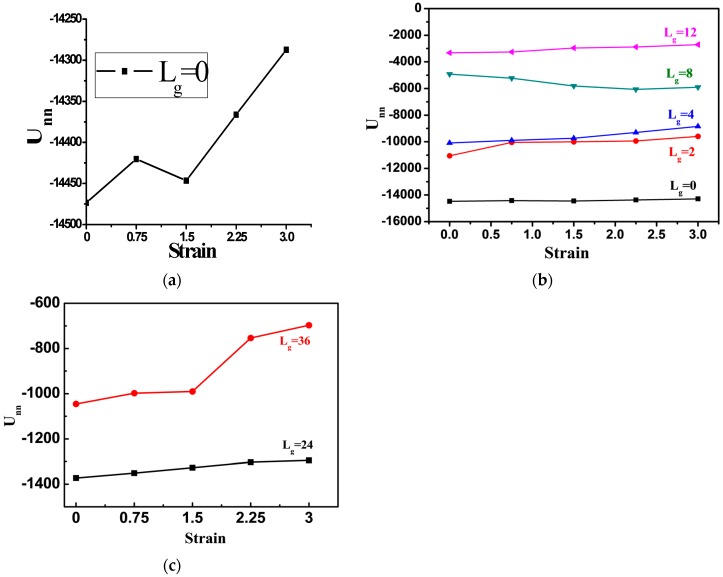
The total interaction energy between NPs $U_{nn}$ as a function of the strain of systems with various grafted polymer chains: (**a**) $L_{g}=0$; (**b**) $L_{g}=0,2,4,8,12$; and (**c**) $L_{g}=24,36$.

To better quantify the deformation process, we calculate the total interaction energy between NPs during tensile process, as shown in Figure 7. For the cases of the bare NPs or NPs grafted with chains $L_{g}=2$, the total interaction energy between NPs is slightly decreased, corresponding to the snapshots in Figure 5a,b. Namely, during the tensile deformation, the tight structure formed by NPs is broken up only slightly away from each other. When the grafted chain length is extended to $L_{g}=4$, the variation trend of the total interaction energy is similar to that of the bare NPs. As suggested by the corresponding snapshot, the sheet-like structure formed by the NPs is extended further away from each other, resulting in a monotonic increase of the interaction energy. However, in the case of the grafted chain length equal to $L_{g}=12$, the total interaction energy is increased during the tensile deformation, implying the break-up of the NPs agglomerates. For the much longer grafted chain length such as $L_{g}=24$ and 36, the total interaction energy between the NPs decreases more significantly, as shown in Figure 7c.

In fact, we have observed that the strongest mechanical reinforcement is obtained when the grafted NPs self-assemble into the sheet structure. Based on the research work carried out by Moll et al. [13], a maximum stress occurs at the moderate strain, followed by the decrease of the stress as a function of the strain, which is also referred to the “stress overshoot”. The occurrence of the maximum stress is to verify the existence of a solid-like structure, which is often observed in those systems with a percolated structure network rather than a well-dispersed structure. Here we also want to probe this issue, by investigating the following three systems such as $L_{g}=2$, $L_{g}=4$ and $L_{g}=8$, and stretch along the y direction to a large strain by keeping the volume unchanged, as displayed in Figure 4d. Obviously, the stress reaches the maximum value, or an overshoot when the strain is equal to 10 for the grafted chain length $L_{g}=4$ with the sheet structure, while only a stress plateau occurs for the cases of $L_{g}=2$ and $L_{g}=8$ even at the large strain. This also supports the fact that the sheet structure gives the best mechanical reinforcement performance. The decline of the stress could result from the break-up of the percolated network structure of the sheet structure.

**Figure 8:**
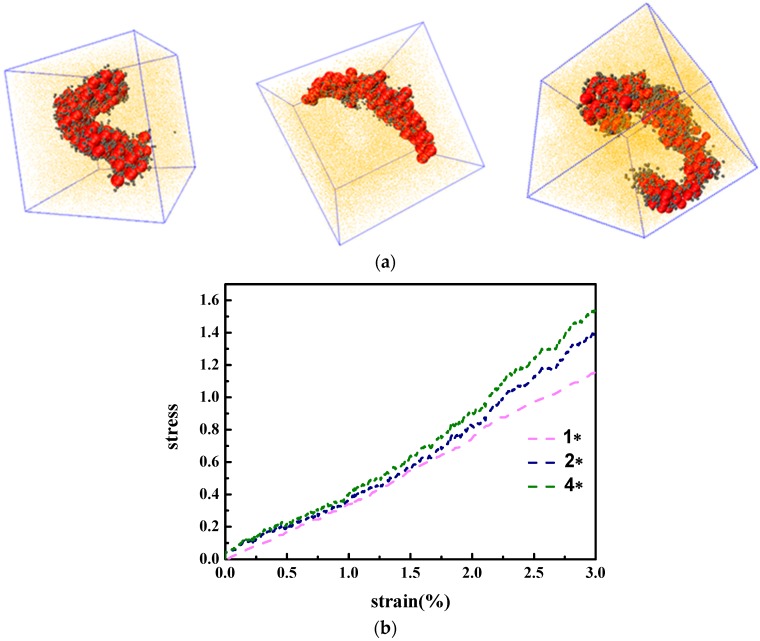
(**a**) Snapshots of PNC systems twice (**middle**) and four (**right**) times the initial system, here the grafted polymer chains are represented by the grey beads, and the polymer matrix is represented by the yellow points while the NPs are denoted by the red spheres; (**b**) stress–strain plots of PNC systems twice and four times the initial system.

Finally, in order to eliminate the effect of the periodic boundary, we demonstrated the finite size effects of the simulation box on the obtained structures and the corresponding mechanical response. We multiplied the whole system by both twice and four times the initial system. After equilibrium of the same conditions, we obtained the self-assembly structure of nanoparticles, as is seen in Figure 8a. It is clearly illustrated that at both conditions, the nanoparticles self-assemble into a cylinder-shaped structure, indicating that our results regarding the system size is not necessarily modified. As for the mechanical properties, we performed a tensile process on both systems and the results are shown in Figure 8b. The stress–strain behavior does not show any obvious shift. This means that our boundary conditions and system size choice is reliable.

### 3.2. Effect of the Flexibility of the Grafted Polymer Chain

In this part, we shift our attention to vary the grafted chain flexibility, which receives little attention about its influence on the relation between the morphology and the mechanical properties. By choosing the grafted chain length $L_{g}=4$, 8, 12, 24 and 36, we introduce the angle potential to model the semi-flexible grafted polymer chain. The potential of angles can be described as follows:

(4) $$E=K{\left(\vartheta -\vartheta_{0}\right)}^{2}$$

in which $\vartheta_{0}$ is the equilibrium value of the angle, and K is the prefactor demonstrating the stiffness of the bond. Here, we set K=50 and $\theta_{0}=180^{{}^{\circ}}$, and overlap grafted polymer beads by 0.25σ with each other, leading the persistence length of the grafted chains to be approximately 4σ.

**Figure 9:**
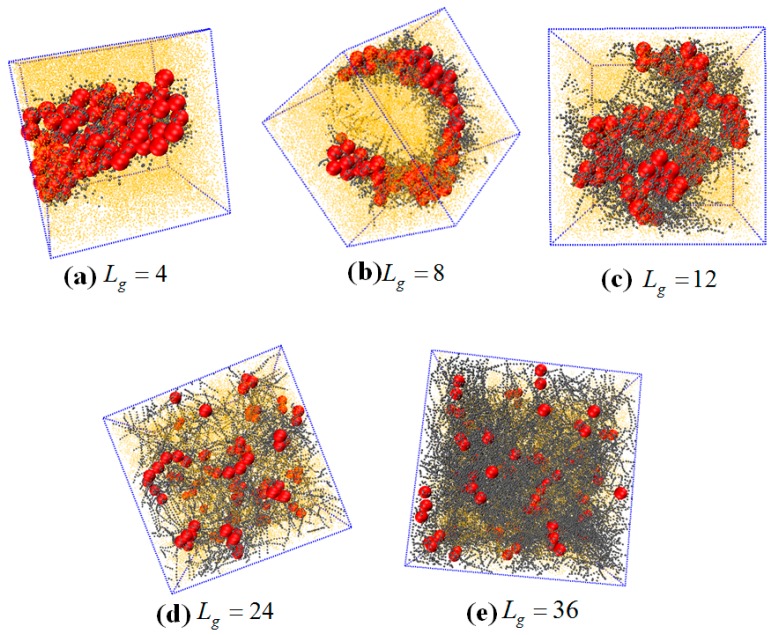
The self-assembly structures of NPs with semi-flexible grafted chains at various grafted chain lengths: (**a**) $L_{g}=4$; (**b**) $L_{g}=8$; (**c**) $L_{g}=12$; (**d**) $L_{g}=24$; and (**e**) $L_{g}=36$. The number of the grafted polymer chains on one NP is fixed to be six, represented by the grey beads. The length of the polymer matrix is set to 100, represented by the yellow points. The NPs are denoted by the red spheres.

**Figure 10:**
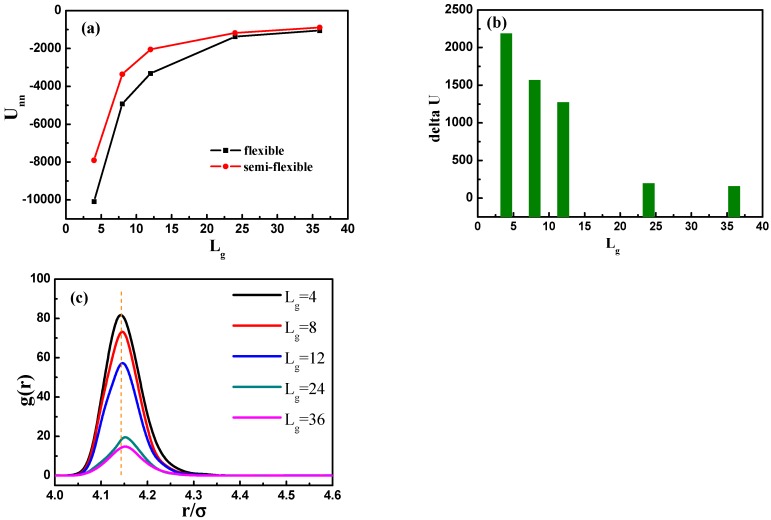
(**a**) The total interaction energy of NPs $U_{nn}$ as a function of grafted chain length $L_{g}$ for systems with flexible and semi-flexible grafted polymer chains; (**b**) the total interaction energy difference delta U between flexible and semi-flexible grafted polymer chains with respect to the grafted chain length $L_{g}$; and (**c**) the radial distribution function of NPs for various grafted chain lengths.

The self-assembled equilibrium structure for various grafted chain length is shown in Figure 9. In the case of $L_{g}=4$, a sheet structure is obtained similar to the case of the flexible grafted chain. For $L_{g}=8$, the NPs self-assemble into a more ordered circle-like string structure. For $L_{g}=12$, 24 and 36, the grafted NPs self-assemble to form short string structure, dispersed and well-dispersed states, respectively, which are similar to the cases with flexible grafted chains. We further calculate the total interaction energy between the NPs as a function of the grafted chain length in Figure 10a. It clearly shows that for the semi-flexible grafted polymer chains, the total interaction between NPs becomes much weaker, compared to the case of the flexible grafted polymer chain at each grafted chain length. The underlying reason is that the semi-flexible grafted polymer chains tend to be extended into a larger corona around each NP, because the semi-flexible polymer chains are less likely to fold to become the coil configuration, making the NPs more difficult to contact with each other and thus resulting in relatively small total interaction energy between NPs. Relevant previous research [16] has shown with aid of experiments that when flexible grafting chains are shifted to rigid chains, the effect of increasing the length of grafted chains on increasing the dispersion of nanoparticles is reduced. In order to better illustrate this, we calculate the difference of the total interaction energy among NPs between the flexible and semi-flexible grafted polymer chains in Figure 10b. For the semi-flexible grafted polymer chains, the dispersion state of NPs is improved with $L_{g}$, which, however, is not as obvious as the case of the flexible grafted polymer chains. Moreover, when the grafted polymer chains become long enough, such as $L_{g}=24$ and 36, the difference between the total interaction energy among NPs becomes not so obvious by comparing the flexible and semi-flexible cases, because in this situation the effect of the chain length surpasses the chain configuration.

In addition, we calculate the radial distribution function of the NPs in Figure 10c, and it is rather clear that the density of the other NPs around each NP is largely reduced with the increase of grafted chain length. It is noticeable that the position of each peak is slightly moved to a large inter-particle distance as the grafted chain length becomes longer.

**Figure 11:**
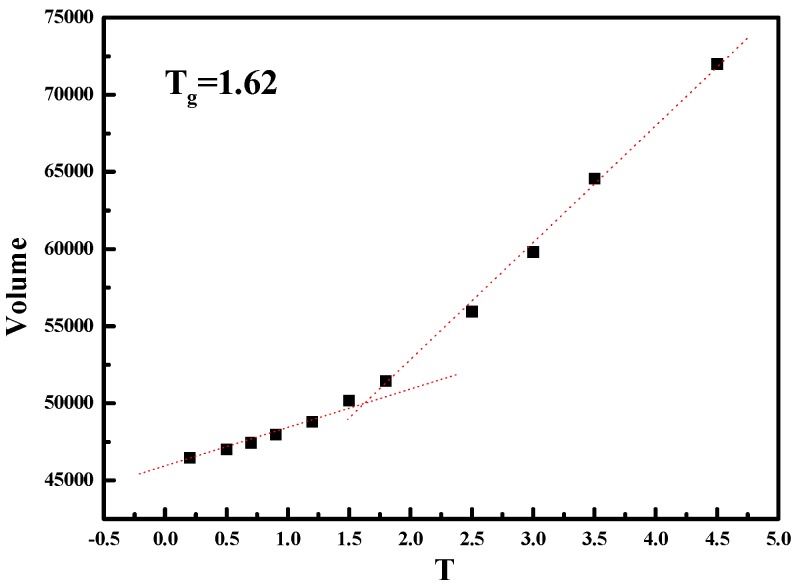
The glass-transition temperature of PNCs with semi-flexible grafted chains when the length of grafted chains equals $L_{g}=4$.

We then characterize the mechanical properties of these five simulated systems with the NPs grafted with semi-flexible polymer chains. It should be noted that our system with semi-flexible grafted chains is still under melting state with temperature T=5.0, and thus the stress–strain behavior is absolutely observed well above glass-transition point, as shown in Figure 11.

**Figure 12:**
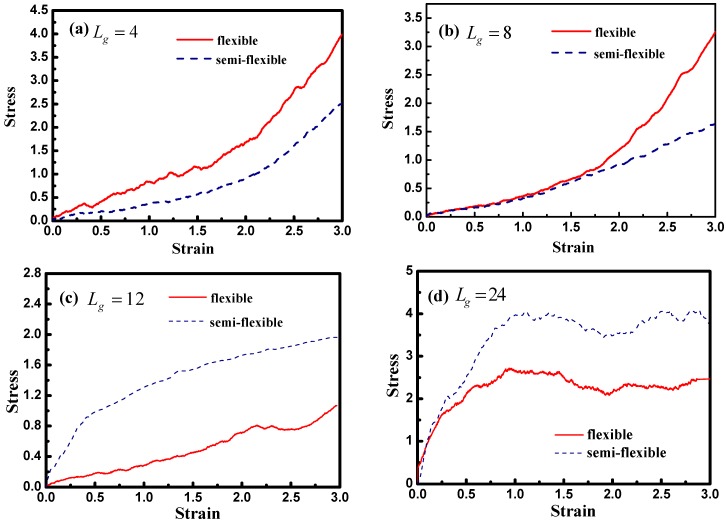

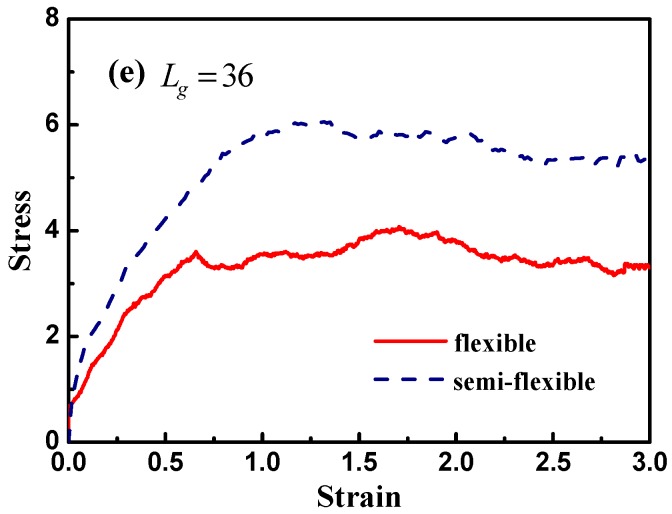
The stress–strain curves for systems containing flexible (**red** line) or semi-flexible (**blue** line) grafted polymer chains with various grafted chain lengths: (**a**) $L_{g}=4$; (**b**) $L_{g}=8$; (**c**) $L_{g}=12$; (**d**) $L_{g}=24$; and (**e**) $L_{g}=36$. Note that there is no interfacial chemical cross-linking between grafted and matrix polymer chains.

**Figure 13:**
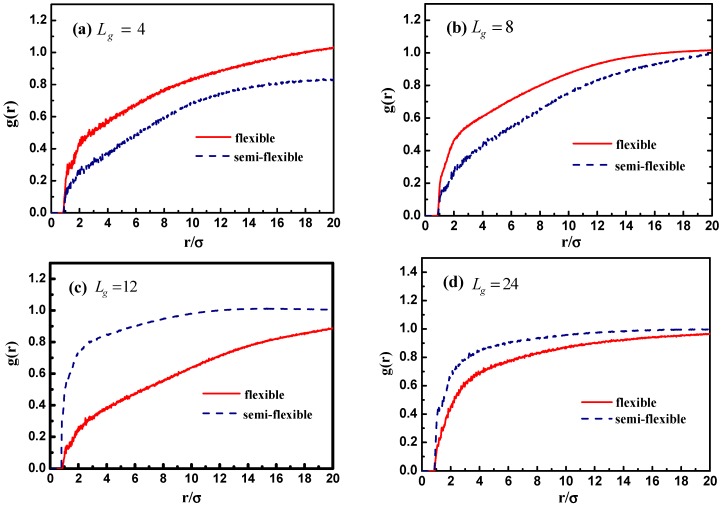

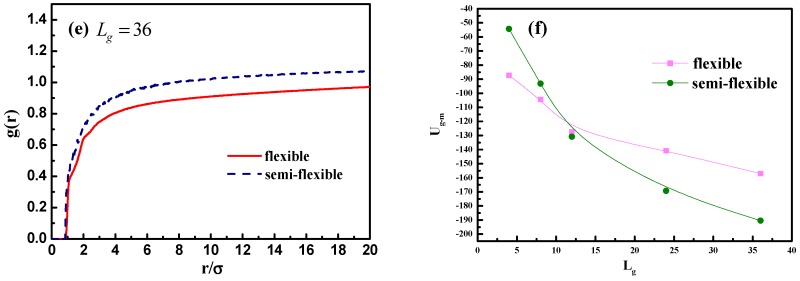
The radial distribution function of the matrix polymer chains around each grafted polymer chain bead (flexible and semi-flexible) for various grafted chain lengths: (**a**) $L_{g}=4$; (**b**) $L_{g}=8$; (**c**) $L_{g}=12$; (**d**) $L_{g}=24$; and (**e**) $L_{g}=36$. (**f**) The interaction parameter between grafted chains and matrix polymer chains. The pink line denotes flexible grafted chains while the green line denotes the semi-flexible grafted chains.

Figure 12 demonstrates the stress–strain curve with different grafted chain length, and we compare the stress–strain curve between semi-flexible grafted chains and flexible grafted chains. It can be seen that for $L_{g}=4$ and 8, a much better stress–strain curve occurs for the case of the semi-flexible grafted polymer chains, compared to the case of the flexible grafted polymer chains, at longer grafted chain length such as $L_{g}=12$, 24 and 36, however, this trend becomes contrary. We infer that at long grafted chain length, the physical inter-locking interaction and wetting between the matrix and grafted polymer chains account for this phenomenon. To better verify our assumption, we calculate the radial distribution function of the polymer matrix beads around each grafted chain polymer bead as shown in Figure 13. The result is consistent with the observation of the stress–strain behavior; namely, at short grafted chain length the density of the polymer matrix beads around the semi-flexible grafted polymer chains is smaller than that of the flexible grafted polymer chain. While at long grafted polymer chain length, the semi-flexible grafted polymer chains become wetter by the polymer matrix compared to the flexible grafted polymer chains. This transition point happens when the grafted chain length increases from $L_{g}=8$ and $L_{g}=12$, as indicated by the radial distribution function shown in Figure 13, which agrees with the transition of the stress–strain behavior versus the grafted chain length. This result just implies that the semi-flexible grafted polymer chain should be long enough to become inter-locking and deeply wet with the surrounding polymer matrix, which is consistent with the experimental observation that the thermo-mechanical properties of PNCs can be critically influenced by polymer–NPs wetting behavior [23]. In order to quantitatively determine the scale of how much semi-flexible polymer and flexible polymer become compatible with each other regarding wetting, we hereby calculated the interaction parameter between the grafted polymer chains and matrix polymer chains with conditions of both flexible grafted chains and semi-flexible grafted chains. As shown in Figure 13f, the flexible grafted chains show a better wetting with matrix at shorter lengths, while the semi-flexible grafted chains show a better wetting at longer chain lengths.

**Figure 14:**
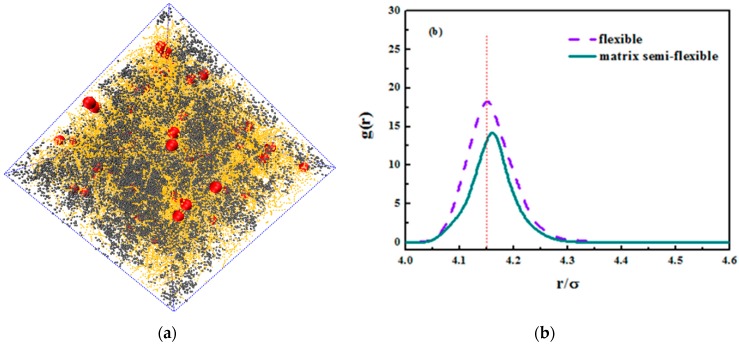

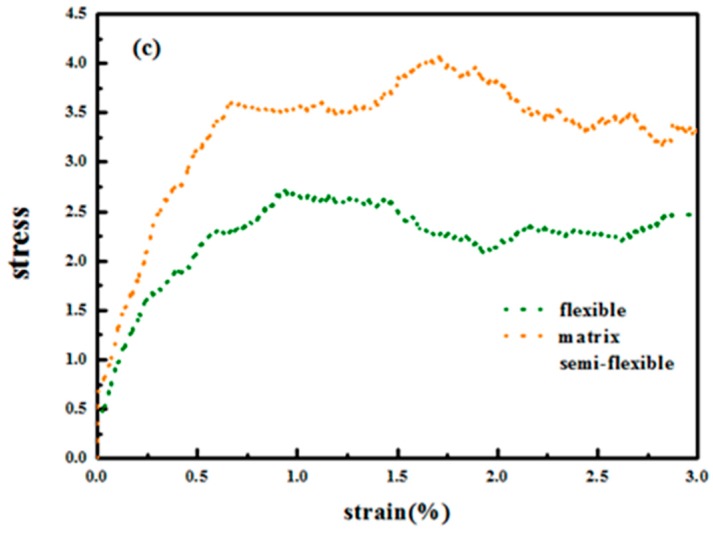
(**a**) The snapshot of grafted nanoparticle-filled polymer system. Note that the red spheres are nanoparticles, the yellow dots are matrix polymer chains and the grey dots are grafted chains; (**b**) The radius distribution function of nanoparticles for both flexible chains and semi-flexible matrix chains; (**c**) The stress-strain plot of systems for both flexible chains and semi-flexible matrix chains.

We hereby demonstrate the situation where the matrix polymer chains are semi-flexible and the grafted chains are flexible merely as a reference. We concentrated on the grafted chain length of $L_{g}=24$, and an equally nice dispersion is seen in the snapshot in Figure 14a. In addition, we compared the radius distribution function of nanoparticles in both semi-flexible matrix chains and flexible matrix chains. The nanoparticles in semi-flexible matrix chains demonstrate a better dispersion that those in flexible matrix chains, which can validate the former idea that the entanglement between grafted chains and matrix chains promotes the dispersion of nanoparticles. As the matrix chains become semi-flexible, it is less likely that they curl into a coil and the surface area increases, leading to a better interaction with grafted chains. As for its tensile response, the comparison is shown in Figure 14c. It is not hard to see that the rigid matrix chains enhance the tensile properties. The matrix chains here undertake most of the stress especially during plateau deformations. As matrix chains become more rigid, they are less likely to slide with each other under tensile and instead they orient with tensile direction and the covalent bonds take up most of the stress. The covalent bonding interaction is larger than the van der Waals’ interaction, so the tensile strength is relatively large for semi-flexible chains.

**Figure 15:**
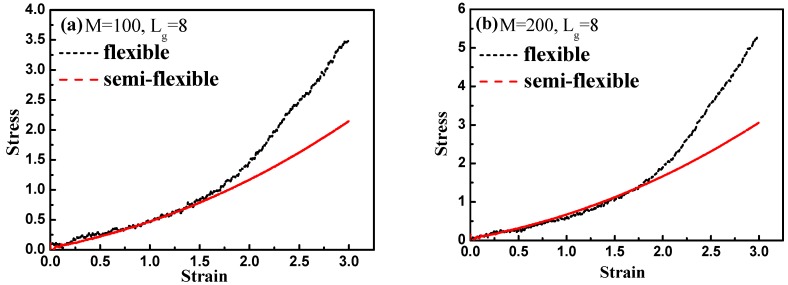

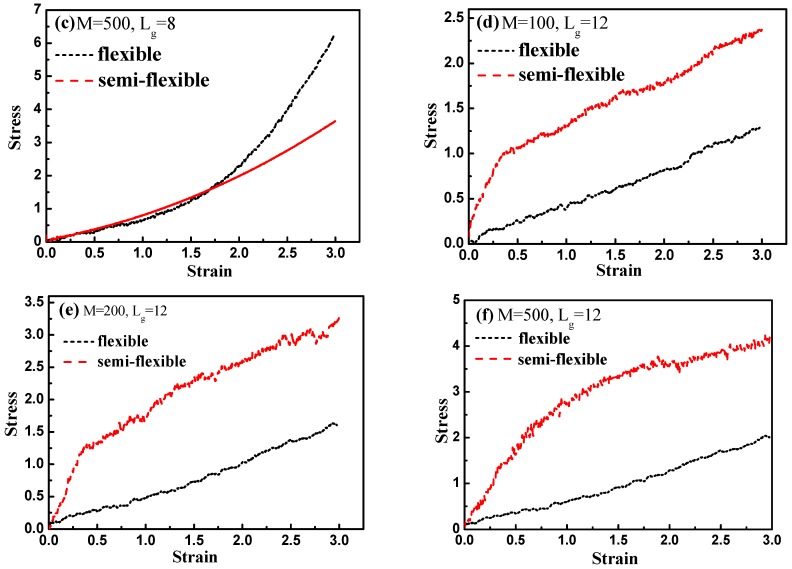
Comparison of the stress–strain curve between flexible and semi-flexible grafted polymer chains by varying the grafted chain length $L_{g}$ and the number of interfacial chemical couplings M: (**a**) $L_{g}=8$, M=100; (**b**) $L_{g}=8$, M=200; (**c**) $L_{g}=8$, M=500; (**d**) $L_{g}=12$, M=100; (**e**) $L_{g}=12$, M=200; and (**f**) $L_{g}=12$, M=500.

Finally, by introducing interfacial chemical cross-linking between grafted and matrix polymer chains, we compare the stress–strain behavior between the flexible and semi-flexible grafted chains systems like $L_{g}=8$ and $L_{g}=12$, and we vary the number of the cross-linked bonds M such as 100, 200, and 500. The way we introduce a cross-linking is by generating a chemical bond between one grafted chain bead and one matrix chain bead. We choose the grafted chains and matrix chains randomly, and a maximum of one cross-linking is generated for each chain. In this case, our system is like the system of rubber. Our results are shown in Figure 15. From Figure 15a–c, it is clearly shown that with the increase of the interfacial cross-linking density, both flexible and semi-flexible systems exhibit a gradually enhanced stress–strain property. For systems with the grafted polymer chain length $L_{g}=8$, flexible grafted chains help to enhance the mechanical property better than semi-flexible grafted chains. However, as for systems with the grafted polymer chain length $L_{g}=12$, an opposite phenomenon is seen. Namely, the semi-flexible grafted polymer chains reinforce more compared to the flexible grafted polymer chains when the length of the grafted chain is long in presence of the interfacial chemical cross-linking. This result is consistent with the observation we find in Figure 12, in which a “transition point” of the stress–strain curve as well occurs from $L_{g}=8$ to $L_{g}=12$ without any interfacial chemical linking.

## 4. Conclusions

In this work, by varying the grafted chain length, we have observed various self-assembled structures formed by grafted NPs in the homopolymer matrix. By comparing the stress–strain behavior of each morphology, we find that the sheet structure possesses the best mechanical reinforcing efficiency, as well validated by the occurrence of the stress overshoot at large strain. Meanwhile, we also simulate the effect of the grafted chain flexibility on the self-assembled morphology and the resulting mechanical properties. Compared to the flexible grafted polymer chain, we observe that the stress–strain behavior of the semi-flexible grafted polymer chains is enhanced greatly at long grafted chain length, attributed to the physical inter-locking and wetting between matrix polymer chains and grafted polymer chains, which has the same trend in the presence of the interfacial chemical bonding between grafted and matrix polymer chains. In general, it is anticipated that this work could provide some guidance on tailoring the mechanical performance of grafted NPs filled polymer nanocomposites, by taking advantage of varying the grafted chain length and flexibility.

## Figures and Tables

**Table 1:** Diameter and mass of simulated particles.

Atom Type	Description	Bead Diameter	Bead Mass
1	Nanoparticles	4	64
2	Virtual surface points	0	0
3	Virtual surface grafted points	0	0
4	Grafted chain end for grafting	1	1
5	Grafted chains	1	1
6	Matrix chains	1	1

**Table 2:** The number of clusters and the number of nanoparticles per cluster with respect to strain change.

Strain	0%	50%	75%	150%	225%	275%	300%
*L*_g_ = 2	1/100	2/50	2/50	2/50	2/50	2/50	2/50
*L*_g_ = 4	1/100	1/100	1/100	1/100	1/100	1/100	1/100
*L*_g_ = 8	1/100	1/100	2/50	2/50	3/33	4/25	5/20
*L*_g_ = 12	1/100	3/33	3/33	3/33	3/33	3/33	3/33
